# RNA-Dependent Protein Kinase Is Essential for 2-Methoxyestradiol-Induced Autophagy in Osteosarcoma Cells

**DOI:** 10.1371/journal.pone.0059406

**Published:** 2013-03-19

**Authors:** Caihong Yang, Kristen L. Shogren, Ribu Goyal, Dalibel Bravo, Michael J. Yaszemski, Avudaiappan Maran

**Affiliations:** 1 Department of Orthopedics, Mayo Clinic, Rochester, Minnesota, United States of America; 2 Department of Orthopedic, Tongji Hospital, Tongji Medical College, Huazhong University of Science and Technology, Wuhan, China; Faculté de médecine de Nantes, France

## Abstract

Osteosarcoma is the most common primary malignant bone tumor in children and young adults. Surgical resection and adjunctive chemotherapy are the only widely available options of treatment for this disease. Anti-tumor compound 2-Methoxyestradiol (2-ME) triggers cell death through the induction of apoptosis in osteosarcoma cells, but not in normal osteoblasts. In this report, we have investigated whether autophagy plays a role in 2-ME actions on osteosarcoma cells. Transmission electron microscopy imaging shows that 2-ME treatment leads to the accumulation of autophagosomes in human osteosarcoma cells. 2-ME induces the conversion of the microtubule-associated protein LC3-I to LC3-II, a biochemical marker of autophagy that is correlated with the formation of autophagosomes. Conversion to LC3-II is accompanied by protein degradation in 2-ME-treated cells. 2-ME does not induce autophagosome formation in normal primary human osteoblasts. In addition, 2-ME-dependent autophagosome formation in osteosarcoma cells requires ATG7 expression. Furthermore, 2-ME does not induce accumulation of autophagosomes in osteosarcoma cells that express dominant negative mutant RNA-dependent protein kinase (PKR) and are resistant to anti-proliferative and anti-tumor effects of 2-ME. Taken together, our study shows that 2-ME treatment induces PKR-dependent autophagy in osteosarcoma cells, and that autophagy could play an important role in 2-ME-mediated anti-tumor actions and in the control of osteosarcoma.

## Introduction

Autophagy is a regulatory event that involves lysosomal degradation of cytoplasmic organelles or cytosolic components [1]. Autophagy occurs due to cellular stress, starvation, hypoxia, cancer or other pathological conditions, and is a multi-step regulatory event involving several signal pathways. Autophagy can be pro- or anti-tumorigenic in nature [2]–[5]. In some instances, autophagy could block cancer cells against drug-induced cytotoxic effects and apoptosis. In other cases, tumor cells can be subjected to autophagic cell death after anti-cancer therapy. Several reports indicate that autophagy may contribute to the survival of established tumors by facilitating chemoresistance during cancer therapy [6]–[10]. On the contrary, autophagy mediates the cytotoxic or cytostatic effect of several anticancer and chemotherapeutic agents suggesting that autophagy could serve as a tumor suppressor event [11]–[14].

Osteosarcoma is a common pediatric tumor which mostly originates in the growing ends of bones and primarily affects children and adolescents. It is the second most common tumor among children who are 15 years of age or younger. Although the overall incidence is low, about 30% of patients diagnosed with osteosarcoma will develop metastatic diseases. Although surgery and chemotherapy have improved the survival in the last decades, a highly successful treatment is not yet available for this disease. 2-Methoxyestradiol (2-ME) is a naturally occurring metabolite of 17β-estradiol and is an inhibitor of tumor cell proliferation in various types of cancer [15]–[21]. 2-ME has been shown to block cell proliferation and induce cell cycle arrest and apoptosis in osteosarcoma cells [22]–[24]. 2-ME-mediated anti-tumor actions require RNA-dependent protein kinase (PKR) in osteosarcoma cells. In this report, we have investigated whether 2-ME actions involve autophagy in osteosarcoma cells.

## Materials and Methods

### Cell Culture

Human osteoblast (HOB) cells and osteosarcoma cells MG63 and KHOS [23]–[26] were cultured in DMEM/F12 media at 37°C and treated with vehicle control and 2-ME as indicated.

### Transmission Electron Microscopy (TEM)

Cells were grown on aclar in tissue culture plates, treated with vehicle or 10 µM 2-ME for 48 hours and then fixed with Trump’s fixative and processed for TEM at Mayo Clinic’s electron microscopy core facility.

### LC3-GFP Assay

MG63 cells were transfected in triplicate with EGFP-LC3B using FuGene-HD transfection reagent (Roche Applied Science, Indianapolis, IN) and treated with vehicle or 10 µM 2ME. Punctate fluorescent staining was visualized by confocal microscopy. The quantitation of punctae was carried out by counting 6 fields for each treatment.

### Western Analysis

Cytoplasmic extracts from vehicle- and 2-ME-treated cells were analyzed by western blot hybridization using anti-LC3-I/II, anti-ATG7 (Cell Signaling Technology, Beverly, MA) and anti-actin (Sigma, St. Louis, MO) antibodies. Quantitation was performed by densitometry and analyzed using Quantity One software (Bio-Rad, Hercules, CA).

### Coimmunoprecipitation Assay

Coimmunorprecipitation was carried out as described [27]. Briefly, cell extracts containing equal amounts of protein were immunoprecipitated using anti-LC3B antibodies, or non-immune IgG. The immunoprecipitated proteins were incubated with protein A Sepharose for 1 hr at 4°C and then washed and analyzed by western blot using anti-p62 and anti-LC3B antibodies (Cell Signaling Technology).

### SiRNA Transfection

siRNA-mediated inhibition of gene expression: Osteosarcoma cells (5×10^4^) in 24-well plates were transfected with a pool of ATG7 siRNAs [i) CCAACACACUCGAGUCUUU; ii) GAUCUAAAUCUCAAACUGA; iii)GCCCACAGAUGGAGUAGCA; iv) GCCAGAGGAUUCAACAUGA] and control non-targeting siRNAs [5′UGGUUUACAUGUCGACUAA3′] as per the manufacturer’s protocol (Dharmacon, Lafayette, CO). Twenty-four hrs after transfection, cells were treated with vehicle control and 2-ME (10 µM). At the end of 24 hrs of treatment, cells will be processed for TEM and western blot hybridization as described above.

### Protein Degradation Assay

Osteosarcoma cells were plated and the following day, 2 µCi of 14C-L-valine was added and incubated for 24 hrs. Then the cells were rinsed and treated with vehicle and 10 µM 2-ME in fresh media. The media were collected at different time intervals and 1 X cell lysis buffer was used to lyse the cells. The radioactivity was determined by precipitating with 10% trichloroacetic acid and counting in a scintillation counter. The protein degradation was determined by the ratio of label in the media to the cells.

### Statistical Analysis

All values are expressed as means+standard error. The data is representative of three independent experiments. Significant differences between groups were determined by Fisher’s protected least significant difference post hoc test for multiple-group comparisons, following detection of significance by one-way analysis of variance (ANOVA). P<0.05 was considered statistically significant.

## Results

### 2-ME Induces Autophagosome Formation in Osteosarcoma Cells

**Figure 1:**
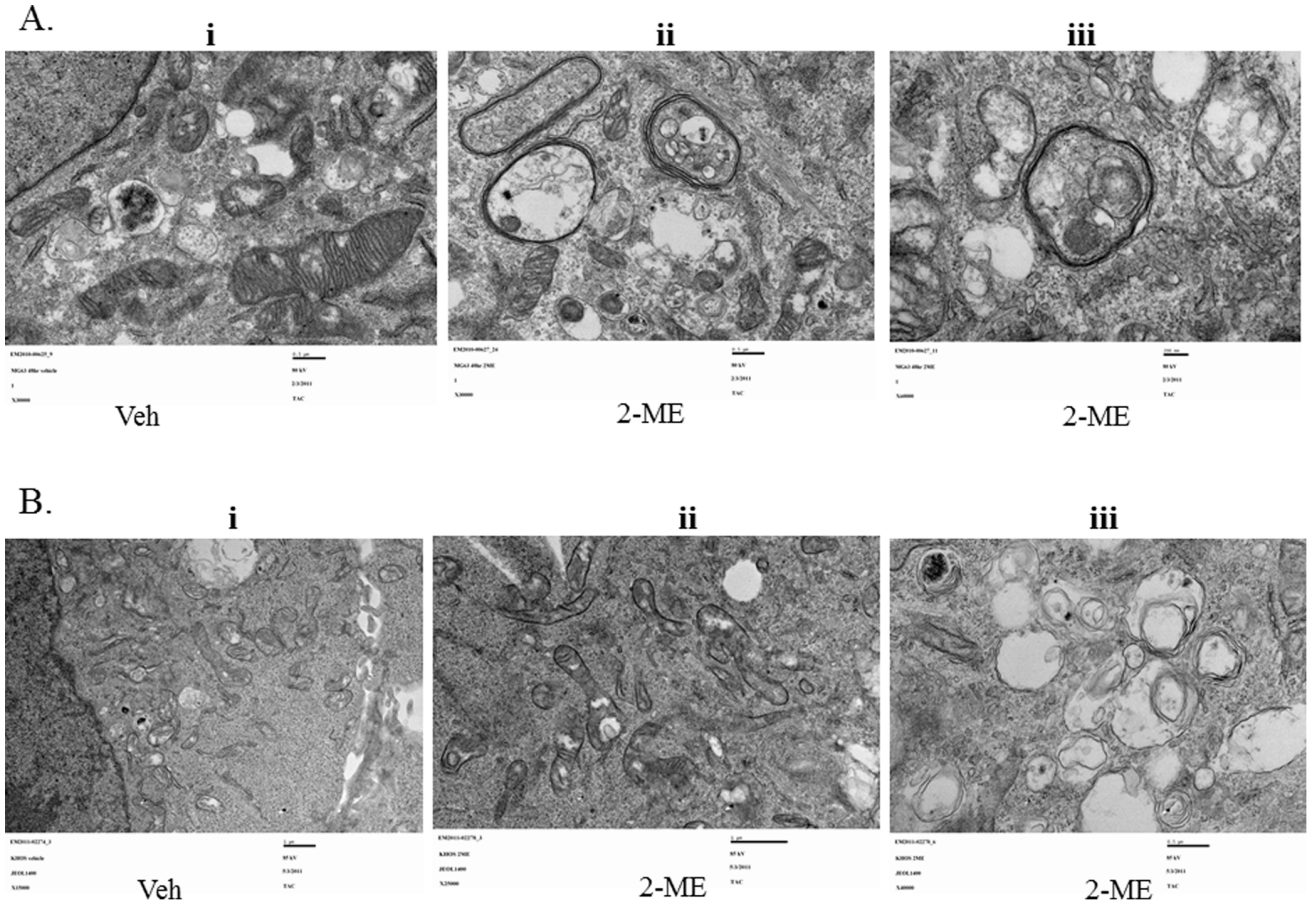
2-ME Induces autophagosome in osteosarcoma cells. Osteosarcoma cells were treated with vehicle (Veh) and 2-ME (10 µM) for 48 hrs and analyzed using TEM. A) TEM images of MG63 cells: Veh 30000X (i); 2-ME 30000X (ii); 2-ME 60000X (iii). B) TEM images of KHOS cells; Veh 25000X (i); 2-ME 25000X (ii); 2-ME40000X (iii).

**Figure 2:**
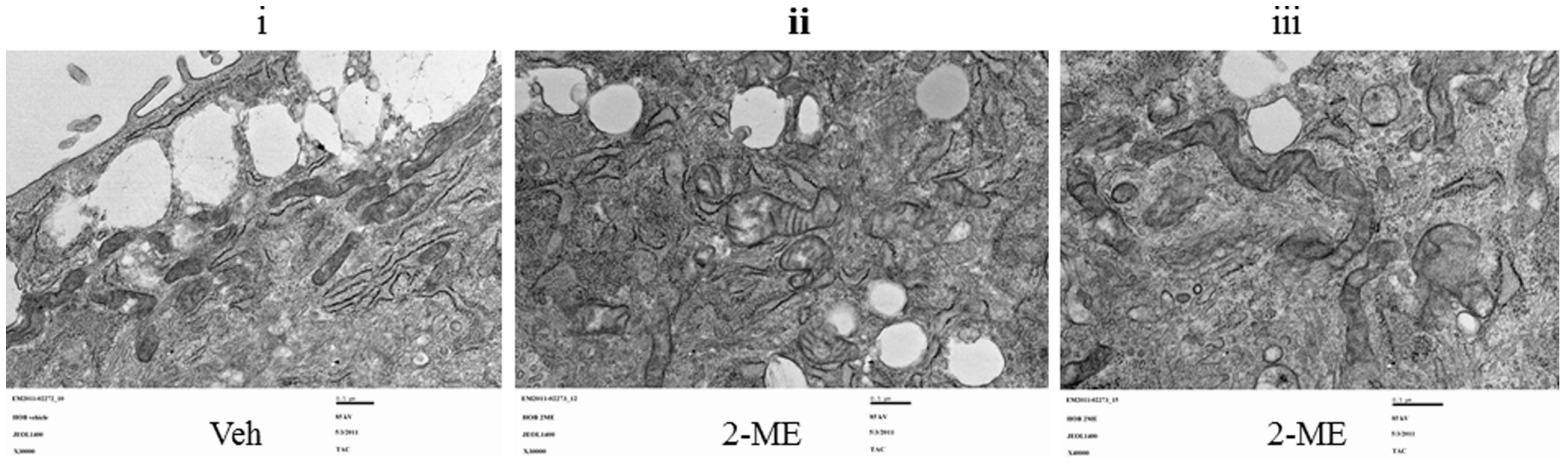
2-ME-does not induce autophagosomes in HOB cells. HOB cells were treated with Veh and 2-ME (10 µM) for 48 hrs and analyzed using TEM. Veh 30000X (i); 2-ME 30000X (ii); 2-ME 40000X (iii).

To determine the effect of 2-ME on autophagy, we have investigated whether 2-ME induces autophagic vacuoles in osteosarcoma cells. The results show that multimembrane vacuoles are formed after 48 hrs in 2-ME-treated MG63 and KHOS osteosarcoma cells, as shown in transmission electron microscopy images (Fig. 1A &1B). The control (vehicle-treated cells) does not show any multimembrane vacuole formation (Fig. 1A &1B). Furthermore, our results show that 2-ME does not induce autophagic vacuoles in primary human osteoblast cells (HOB) suggesting that the effect is specific to tumor cells (Fig. 2).

### 2-ME Induces the Conversion of LC3-I to LC3-II

**Figure 3:**
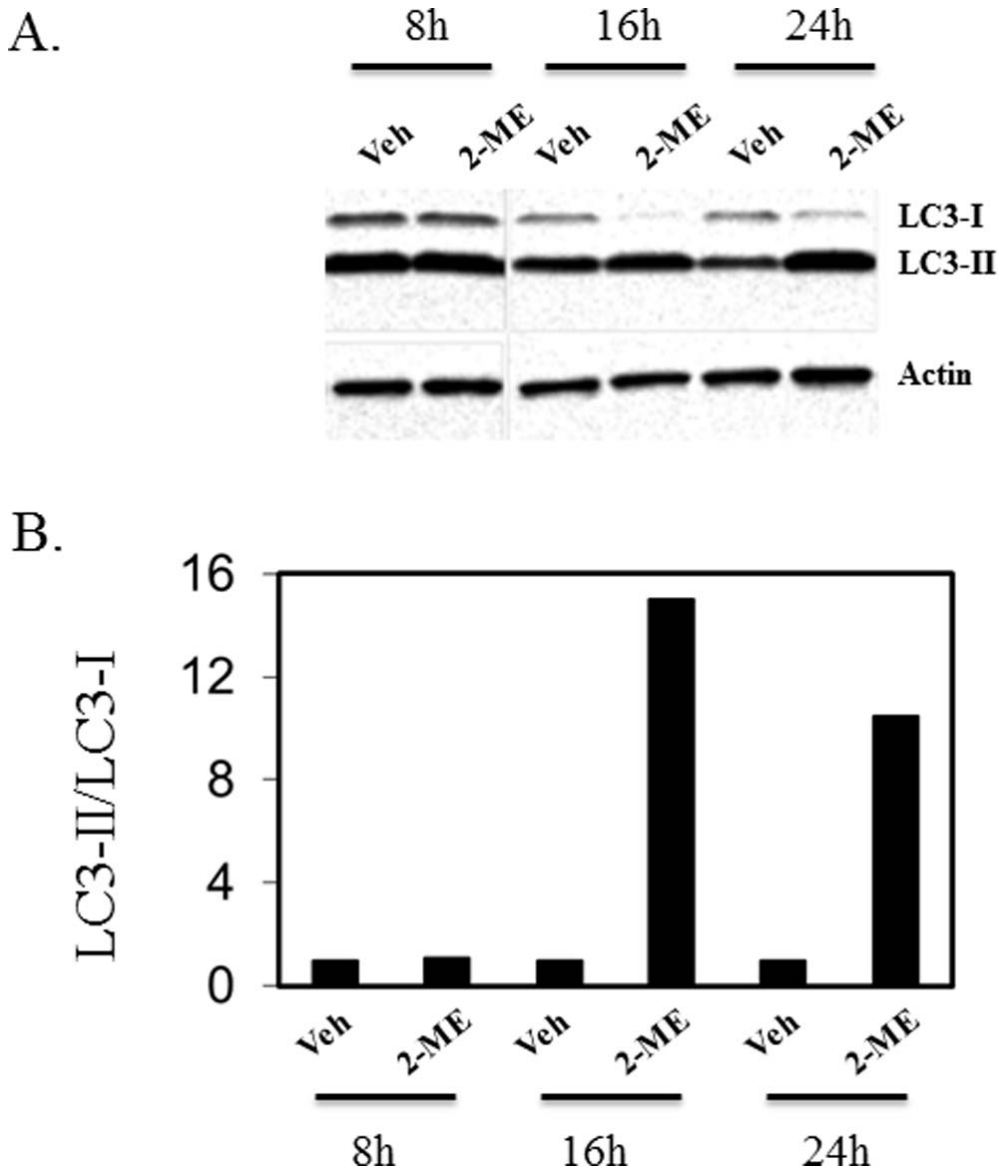
2-ME increases LC3-II/LC3-I ratio in MG63 cells. The cytoplasmic extracts prepared from Veh- and 10 µM 2-ME-treated cells at various time points were analyzed by western blot hybridization using anti-LC3-I/II and anti-actin antibodies. The bands were analyzed by quantitative densitometry. After western blot analysis, the signals from the corresponding bands were quantitated and the ratio of LC3-II over LC3-I was determined after normalization to actin. A) Western analysis; B) Quantitation of the blot.

**Figure 4:**
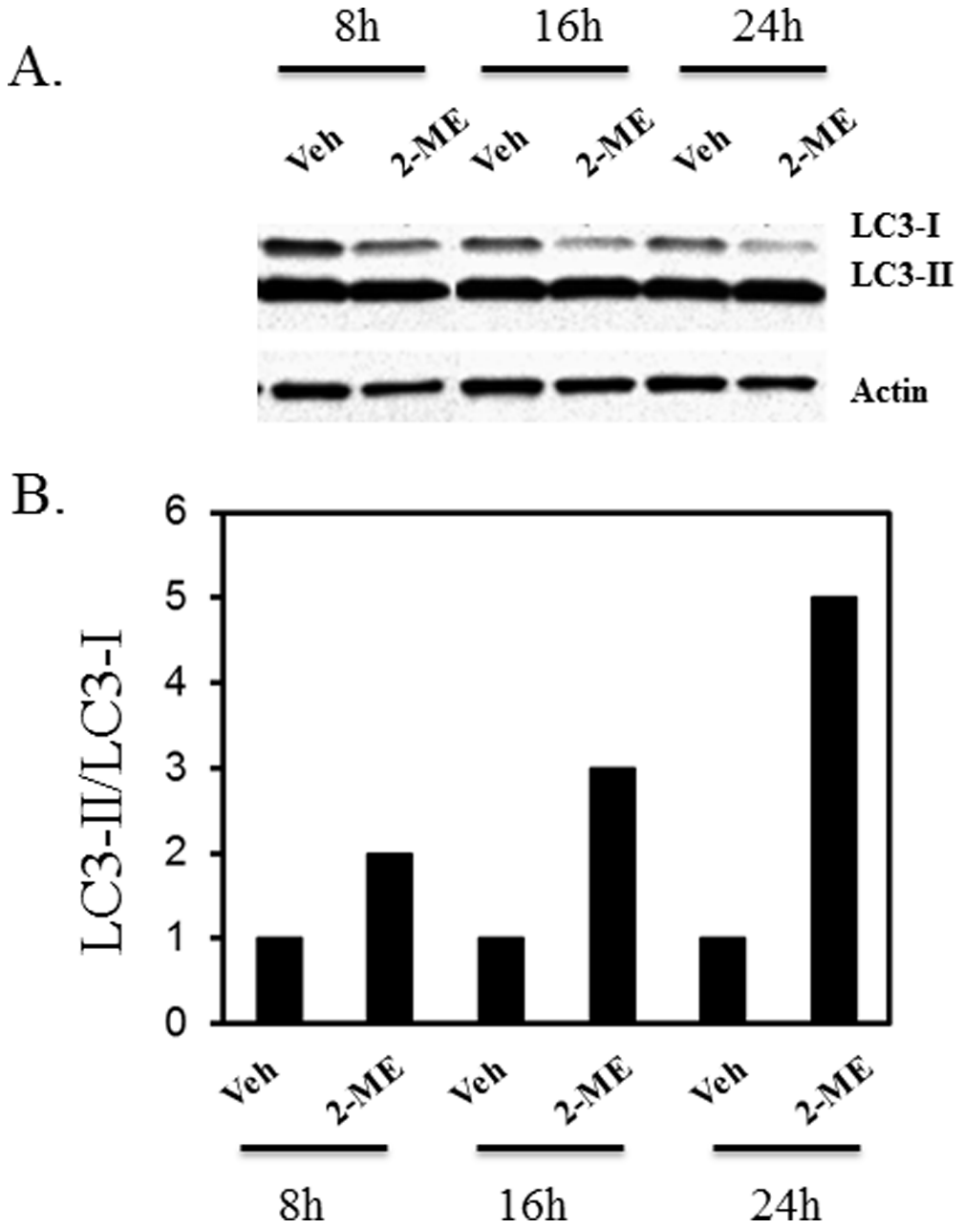
2-ME increases LC3-II/LC3-I ratio in KHOS cells. The cytoplasmic extracts prepared from Veh- and 10 µM 2-ME-treated cells at various time points were analyzed by western blot hybridization as described in Fig. 3. A) Western analysis; B) Quantitation of the blot.

The autophagosome-associated protein, microtubule-associated protein 1 light chain 3 (LC3), has been implicated as a marker of autophagy. During autophagy, LC3 type II, the membrane bound form, increases due to conversion from type I, the cytosolic form. 2-ME treatment induces the accumulation of autophagy marker LC3-II in MG63 and KHOS osteosarcoma cells, compared to the vehicle control (Fig. 3 and 4). The ratio of LC3-II to LC3-I increases in MG63 cells in the presence of 2-ME to 15-fold and 11-fold at 16 and 24 hrs, respectively (Fig. 3). Similarly in KHOS cells, the LC3-II to LC3-I ratio increases at 24 hrs to 5-fold (Fig. 4).

**Figure 5:**
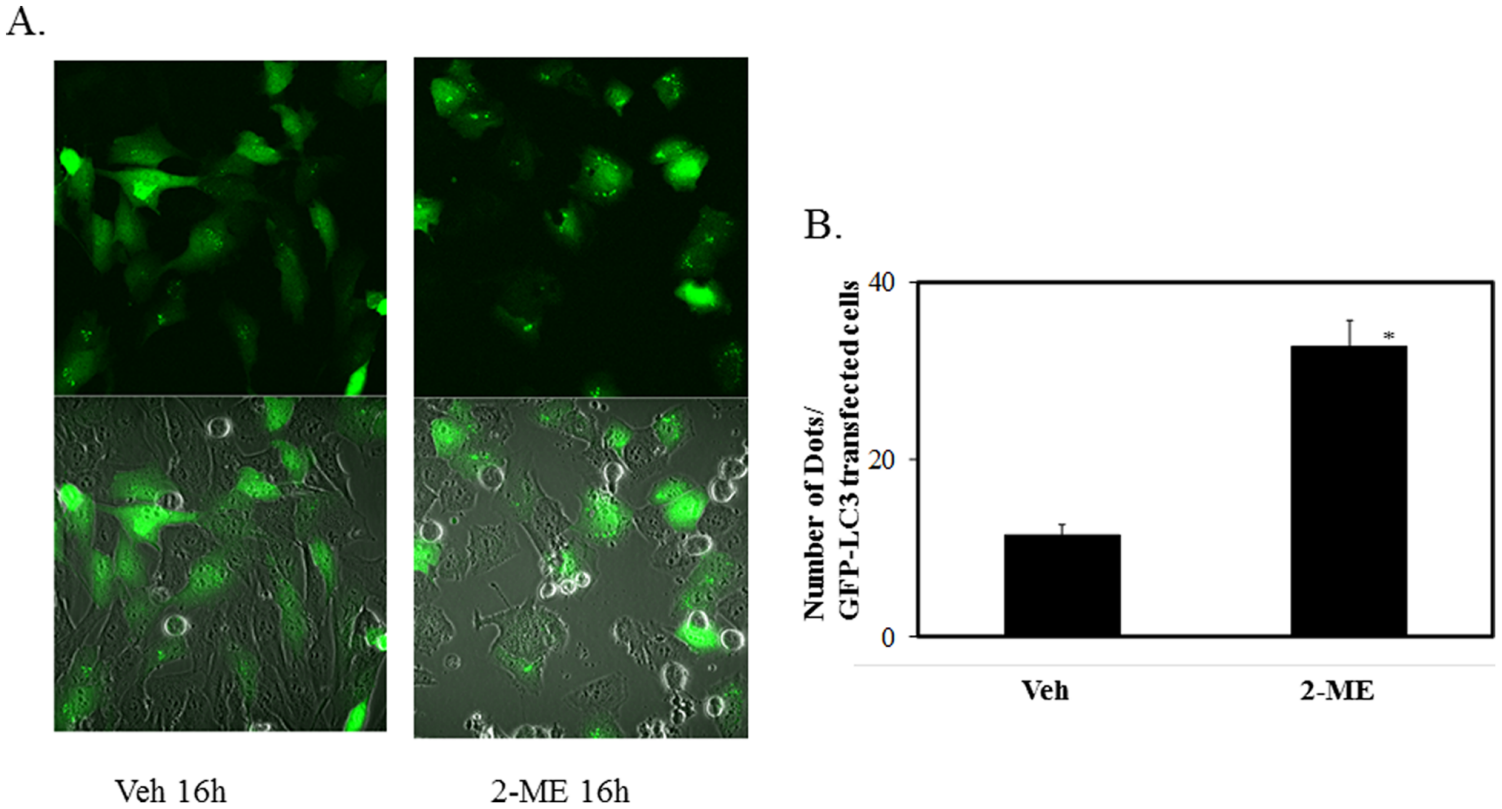
Puncta formation and quantitation in 2-ME-treated cells. LC3-GFP-transfected MG63 osteosarcoma cells were treated with vehicle and 10 µM 2-ME for 16 hrs.GFP-LC3 dots per GFP-LC3-transfected cells were scored after analysis by fluorescence microscopy (A) Upper panel: fluorescence; Lower panel: fluorescence+ VISIBLE. B) Quantitation of Puncta.*P<0.05 compared to Veh.

In addition, we have examined autophagy by following the effect of 2-ME in osteosarcoma cells that have been transfected with GFP-LC3 plasmid constructs. Our results show that 2-ME treatment increased the number of punctae formed, compared to vehicle control at 16 hrs (Fig. 5A). In contrast, vehicle-treated cells showed a diffuse distribution of green fluorescence (Fig. 5A). 2-ME induces the number of punctae by 3-fold (Fig. 5B).

### 2-ME Induces Protein Degradation in Osteosarcoma Cells

**Figure 6:**
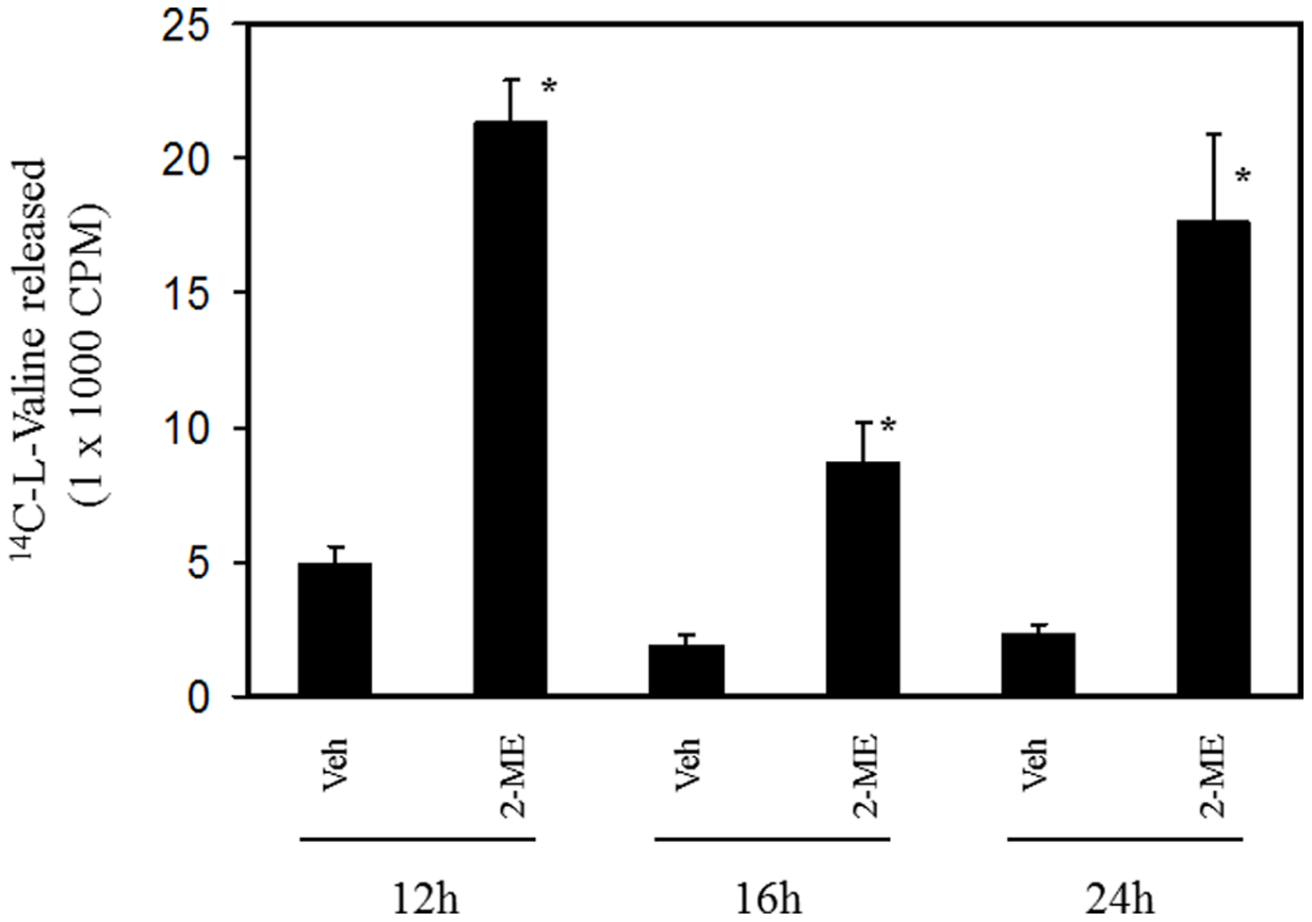
2-ME increases protein degradation in MG63 cells. ^14^C-valine-labeled MG63 cells were treated with vehicle and 10 µM 2-ME for various time points and the protein degradation was analyzed as described in Methods. *P<0.05 compared to Veh.

We have investigated whether induction of autophagosome is accompanied by protein degradation in 2-ME-treated cells. To measure the overall rates of protein degradation, vehicle- and 2-ME-treated MG63 cells were radio labeled by exposure to [^14^C] valine, and the rate of protein degradation was determined by measuring the [^14^C] valine release from the pre-labled proteins into the medium at various time intervals. Compared to the control, the 2-ME treatment increased the protein degradation rate by 4.5-, 4- and 7-fold at 12, 16 and 24 hrs, respectively (Fig. 6).

**Figure 7:**
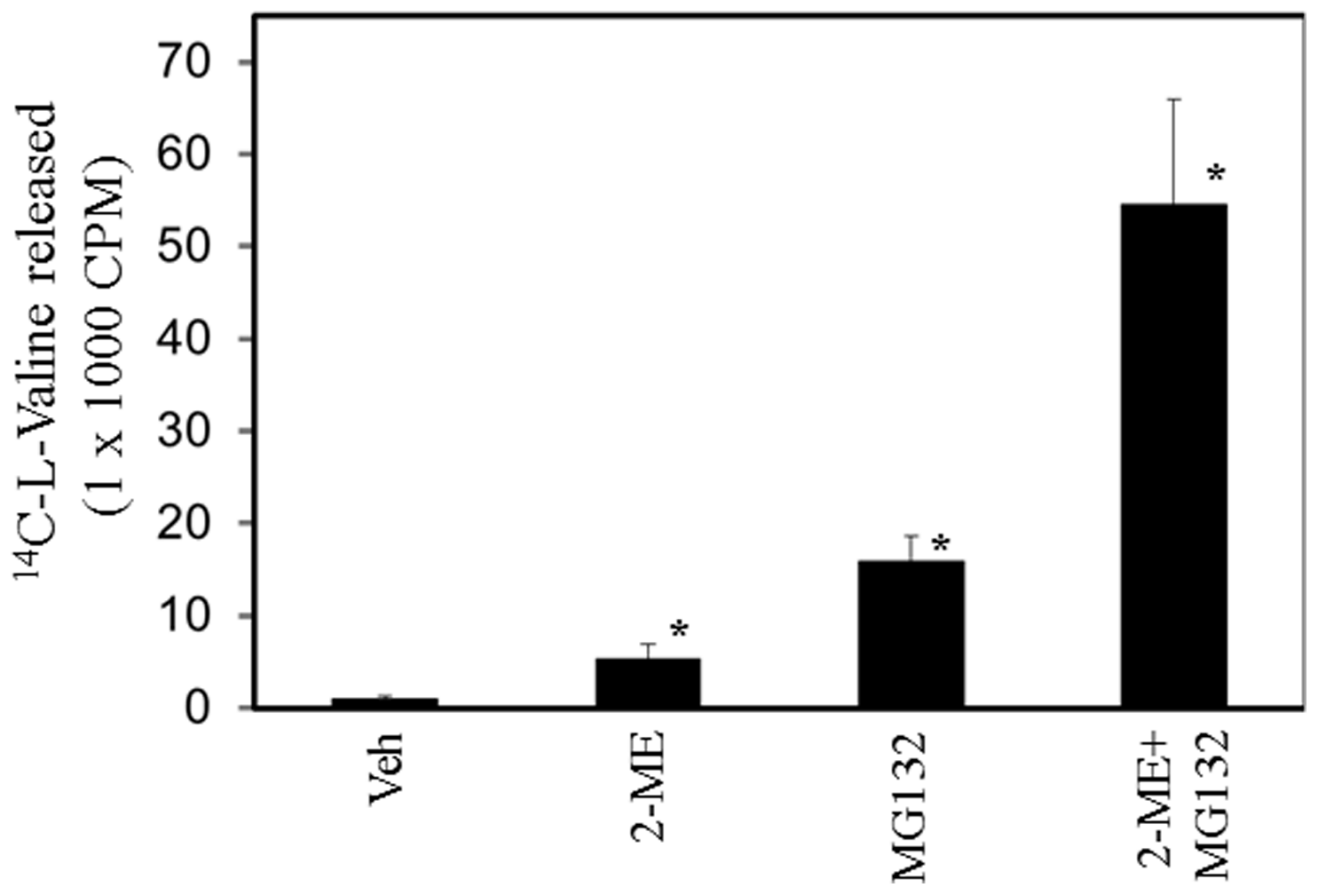
Effect of proteasome inhibitor on protein degradation in 2-ME-treated MG63 **cells.** ^14^C-valine-labeled MG63 cells were treated with vehicle and 2-ME (10 µM) in the presence and absence of MG132 (2.5 µM) for 24 hrs and the protein degradation was analyzed as described in Methods. *P<0.05 compared to Veh.

The effect of 2-ME on protein degradation was further evaluated in the presence of the proteasome inhibitor MG132. As shown in Fig. 7, 2-ME, MG132 and 2-ME plus MG132 combination induced the protein degradation by 5-, 15- and 54-fold, respectively.

### 2-ME Treatment Regulates SQST/P62 Levels and its Association with LC3

**Figure 8:**
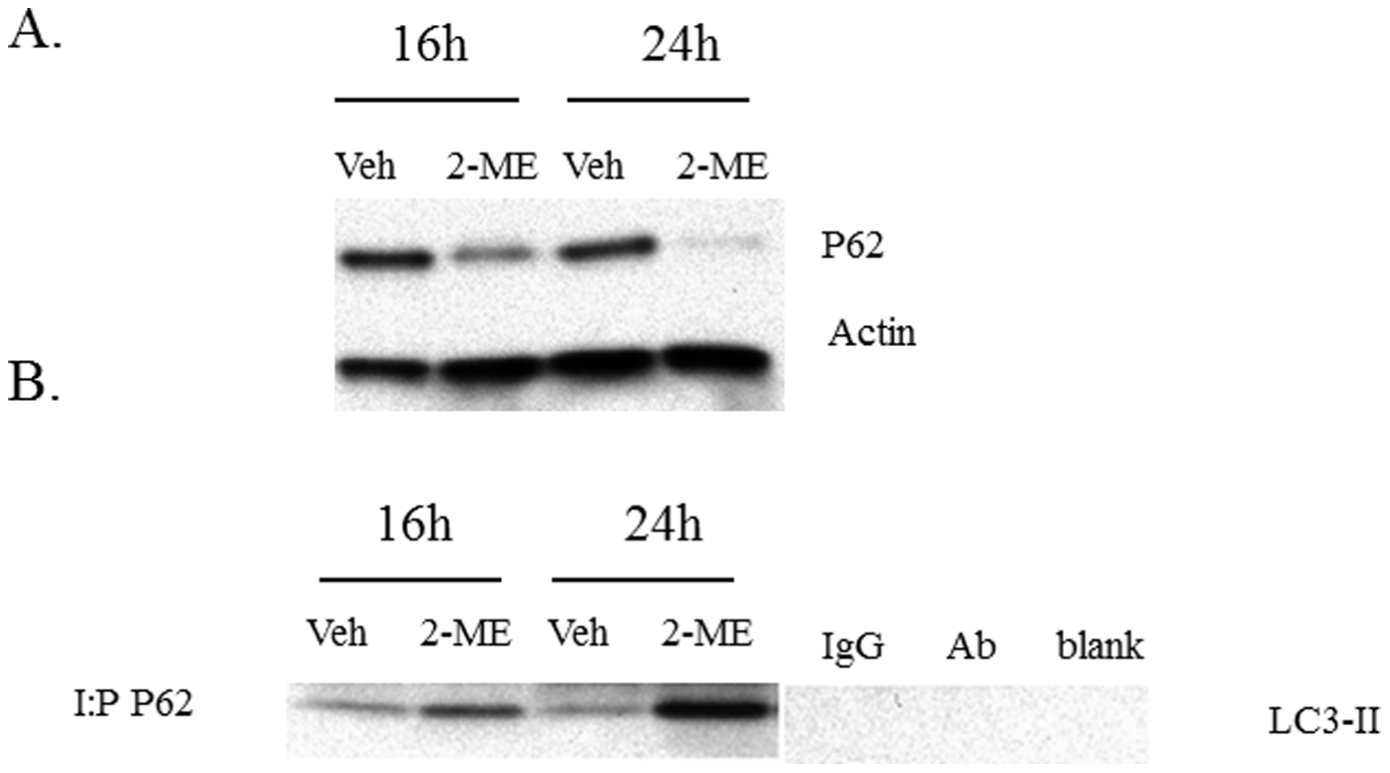
2-ME treatment decreases P62 levels and induces P62 and LC3 interactions. Cytoplasmic extracts were prepared from Veh and 10 µM 2-ME-treated cells at 16 and 24 hrs. A) Analysis by western blot hybridization using anti-P62 and anti-actin antibodies. The signals from the corresponding bands were quantitated and the levels of P62 were determined after normalization to actin. B) Protein extracts were immunoprecipitated with anti-P62 antibodies and analyzed by western blot hybridization using anti-LC3 antibodies. IgG: control with IgG; Ab: control with no antibody; blank: control with no protein extract.

We examined the effect of 2-ME treatment in osteosarcoma cells. Our results show that 2-ME treatment decreases P62 levels at 16 and 24 hrs (Fig. 8A) by 2- and 5-fold, respectively. In order to determine whether p62 interacts with LC3, we carried out co-immunoprecipitation studies. The results show that there is an increased interaction of p62 and LC3-II in 2-ME-treated osteosarcoma cells, compared to the control at 16 and 24 hrs (Fig. 8B).

### SiRNA-mediated Inhibition of ATG7 Blocks Autophagosome Formation in 2-ME-treated Cells

**Figure 9:**
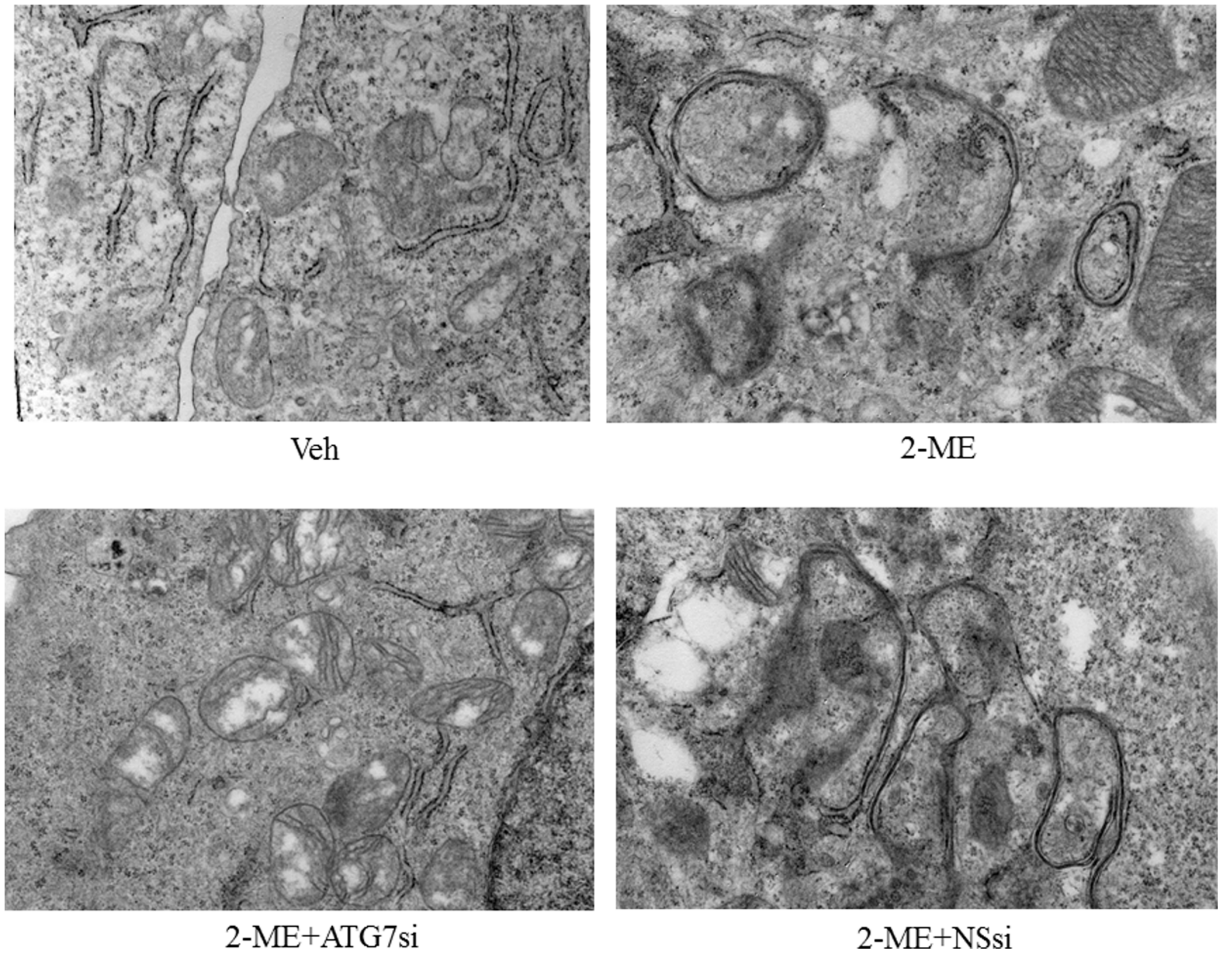
SiRNA-mediated inhibition of ATG7 expression blocks 2-ME-mediated autophagosome formation. MG63 osteosarcoma cells were treated with vehicle (Veh), 10 µM 2-ME, 2-ME plus 100 nM ATG7siRNA (ATG7Si) and 2-ME plus 100 nM Non specific siRNA (NSsi) for 48 hrs and analyzed using TEM.

**Figure 10:**
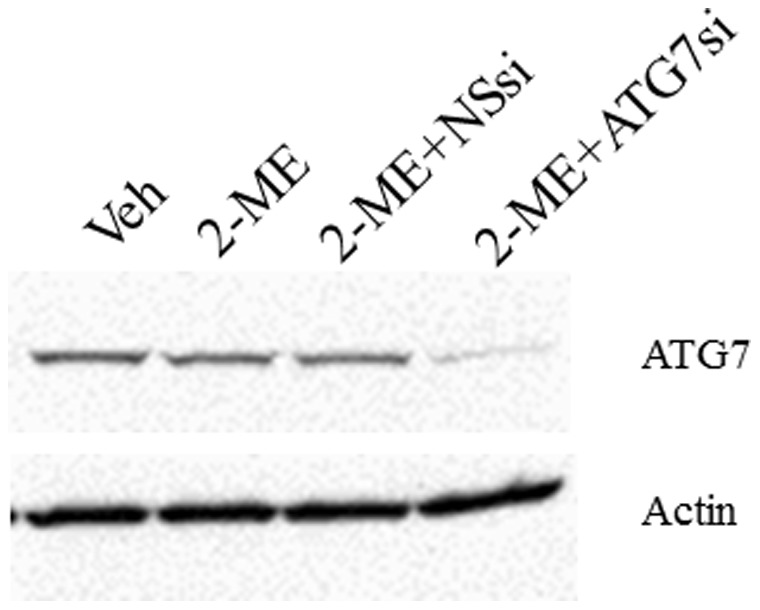
ATG7 siRNA transfection leads to down regulation of ATG7 protein. MG63 osteosarcoma cells were treated with vehicle (Veh), 10 µM 2-ME, 2-ME plus 100 nM ATG7siRNA (ATG7Si) and 2-ME plus 100 nM Non specific siRNA (NSsi) for 48 hrs and analyzed by western blot hybridization using anti-ATG7 and anti-actin antibodies.

ATG7 has been shown to be essential for lysosomal breakdown of organelles, protein and the induction mammalian autophagy. We have examined the effect of 2-ME on autophagosome formation in osteosarcoma cells that have been depleted with ATG7 expression after transfection with siRNAs directed against ATG7 gene. 2-ME treatment does not induce autophagasome formation in osteosarcoma cells transfected with ATG7 siRNAs but induces autophagasome in control cells that have been transfected with non-specific siRNAs (Fig. 9). The data presented in Fig. 10 confirm that siRNA transfection leads to the down regulation of ATG7 protein levels without affecting the control actin levels (Fig. 10).

### Inhibitors of Autophagy Block 2-ME Effects

**Figure 11:**
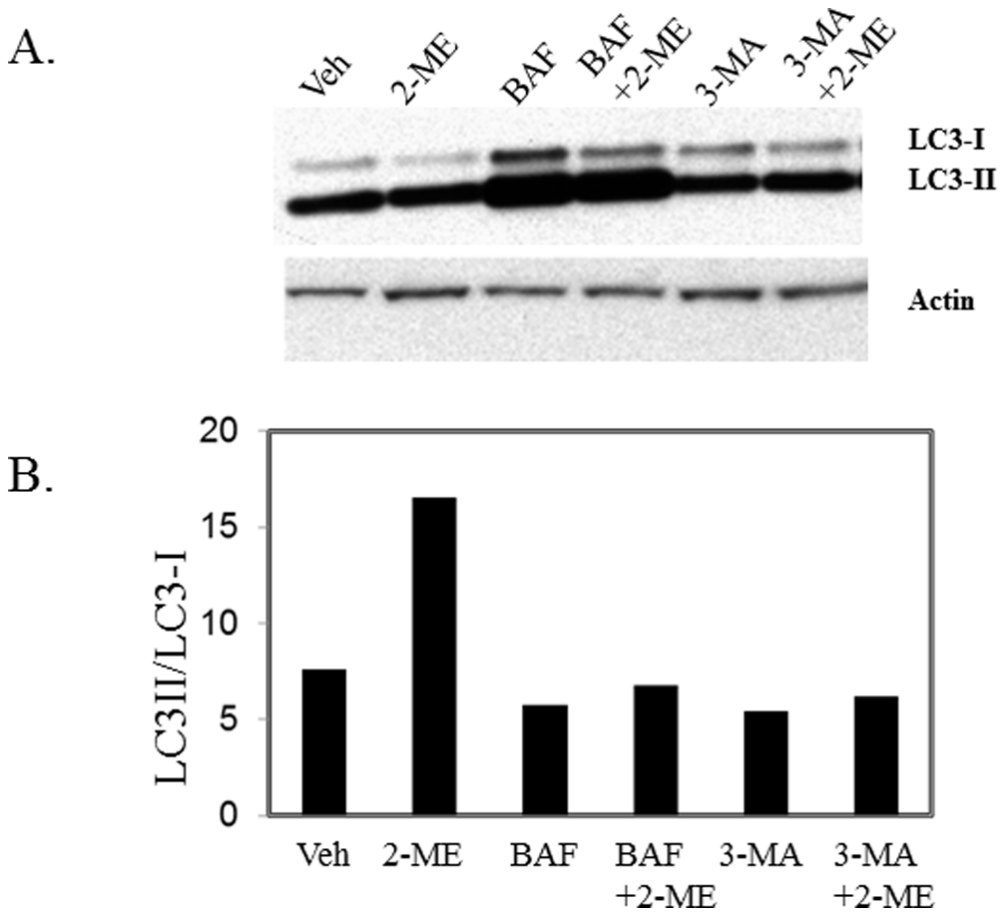
Effect of autophagy inhibitors on 2-ME treatment. MG63 cells were treated with vehicle (Veh) and 2-ME (10 µM) in the presence and absence of 100 nM bafilomycin A1 (BAF) and 20 µM 3-methyladenine (3-MA) for 24 hrs and the ratio of LC3-II over LC3-I was determined as described in Fig. 3. A) Western analysis; B) Quantitation of the blot.

The effects of autophagy inhibitors, bafilomycin A1 and 3-methyladenine (3MA) on 2-ME-mediated conversion of LC3-I to LC3-II and cell death were studied. Both bafilomycin A1 and 3MA blocked 2-ME-mediated conversion of LC3-I to LC3-II (Fig. 11A and 11B).

### 2-ME does not Induce Autophagosome Formation when PKR is Inhibited

**Figure 12:**
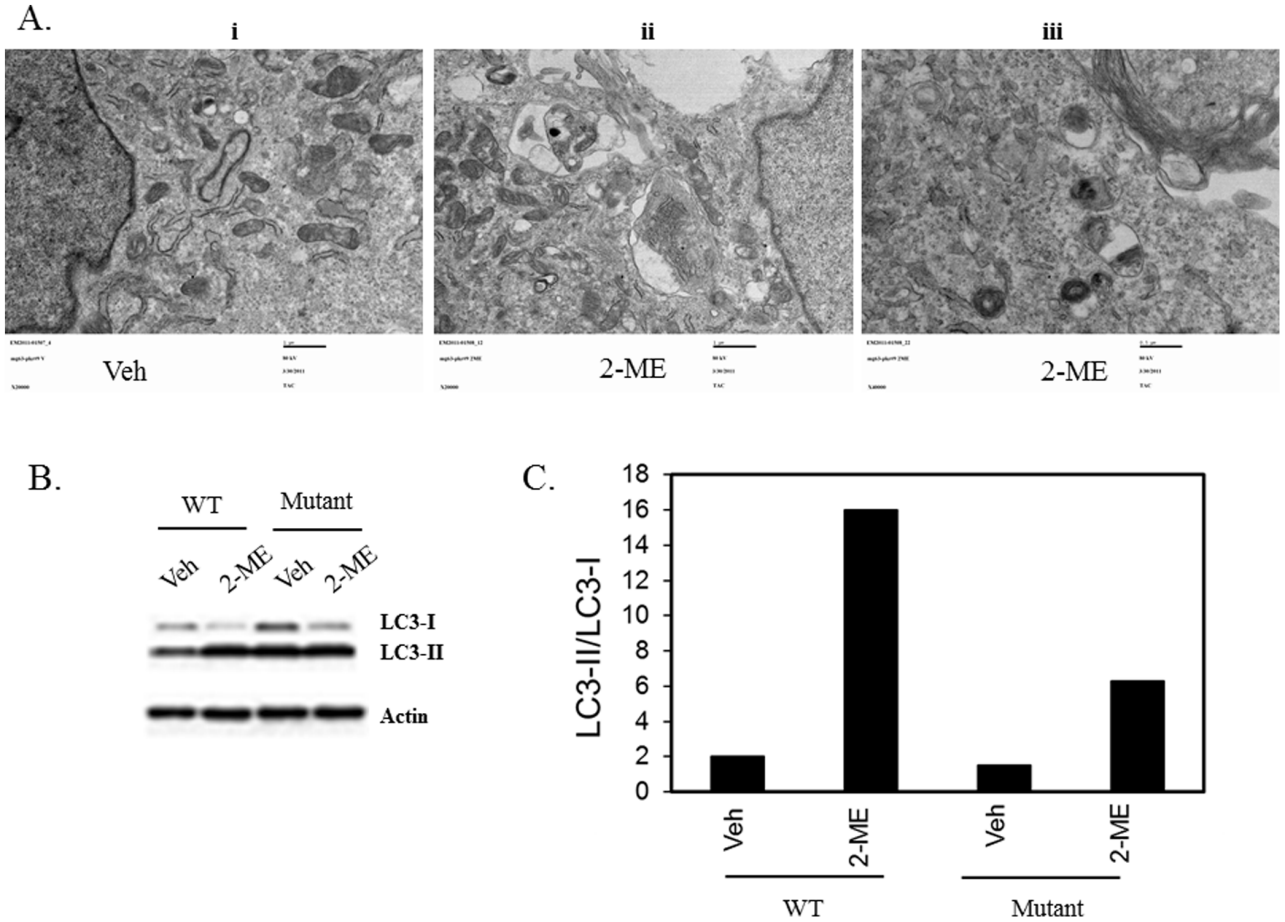
2-ME-mediated effects on autophagy is blocked in cells expressing PKR mutant. PKR mutant expressing MG63 osteosarcoma cells were treated with Veh and 2-ME (10 µM) for 48 hrs and analyzed using transmission electron microscopy. A)TEM images. Veh 20000X (i); 2-ME 20000X (ii); 2-ME40000X (iii); B) Cell extracts were prepared from wild type (WT) and mutant PKR protein expressing MG63 cells at 16 hrs after Veh and 2-ME treatment and the LC3I and LC3 II levels were measured by western analysis as described in Fig. 3. C) Quantitation of the Western blot.

2-ME treatment induces PKR expression and PKR activity in osteosarcoma cells [24]. To determine whether PKR is required for 2-ME-mediated autophagic flux, we have investigated the effect of 2-ME on autophagosome formation and LC3-I to LC3-II conversion, in osteosarcoma cells that have been stably-transfected with trans- dominant mutant PKR cDNAs and are defective for PKR activity and resistant to 2-ME-mediated anti-proliferative effects. 2-ME does not induce autophagosome formation in MG63 cells expressing mutant PKR protein (Fig. 12A). Our results show that the 2-ME-mediated conversion of LC3-I to LC3-II is inhibited in MG63 cells expressing mutant PKR and the LC3-II conversion is decreased to 4-fold compared to 8-fold observed in wild type PKR expressing MG63 cells (Fig. 12B &C).

## Discussion

In this report, we show that 2-ME induces autophagosome formation, autophagy markers and protein degradation, in osteosarcoma cells. 2-ME induces autophagy specifically in osteosarcoma cells, as it does not induce autophagosome formation or markers of autophagy in normal osteoblasts. Our findings also show that 2-ME-mediated autophagy requires RNA-dependent protein kinase (PKR). The molecular mechanism of autophagy in tumor continues to be an important focus in many cancer models. Autophagy is regulated in various cancers, and depending on the context, it has been shown to be either pro-cancer or anti-cancer. Autophagy is induced during therapy by chemotherapeutic drugs and has been shown to mediate the cytotoxic effects of drugs [11]–[14]. Current results reveal that 2-ME-mediated anti-tumor actions in osteosarcoma cells need further investigations in order to fully understand the role of autophagy in osteosarcoma patients.

It has been demonstrated that during autophagy, the LC3 protein is recruited. Also, one of the early key events in autophagy is the conversion of cytoplasmic soluble LC3-I to a lipid-conjugated LC3-II form that associates with the autophagosome membranes [28]–[30]. The autophagic activity is measured biochemically based on the amount of LC3-II accumulation and the formation of cellular autophagosome punctae containing LC3-II [31]. The results presented here show that the conversion of LC3-I to LC3-II is induced by 2-ME treatment in osteosarcoma cells. In addition, it has been demonstrated that in GFP-LC3-transfected osteosarcoma cells, 2-ME increases the punctate form of LC3. The data also demonstrate that osteosarcoma cells transfected with siRNAs for ATG7, do not respond to 2-ME-mediated autophagosome induction, suggesting that 2-ME-mediated effects require ATG7 protein expression. Also, 2-ME does not induce autophagosome formation in normal HOB cells, implying that only cells that respond to anti-growth activities of 2-ME respond to 2-ME-stimulated formation of autophagosomes.

Our results show that increased autophagosome formation is accompanied by increased protein degradation in the presence of 2-ME treatment. We have demonstrated that proteasome inhibitor MG132 enhances 2-ME effects on protein degradation. This observation is in agreement with the published reports which indicate that inhibition of proteasome induces autophagy [32], [33]. While further investigation is necessary, the combined effects of 2-ME and MG132 confirm the link between Ubiquitin-proteosome system and autophagy machinery [32], [33].

The P62 protein, also known as Sequestosome 1(SQST1) has been shown to play a role in the regulation of tumor and autophagy and to aggregate to LC3 on the surface of autophagosomes [34], [35]. Similarly, p62 was also localized to the autophagic compartment and constantly degraded by the autophagy-lysosome system. P62 binds to LC3 and can localize to autophagic compartments, thereby transporting ubiquitinated proteins for degradation. This event is widely used as a common hallmark of autophagy [34], [35]. Our results demonstrate that there is a decrease in p62 expression but an enhanced association of p62 to LC3-II in the presence of 2-ME-treatment in osteosarcoma cells. These 2-ME-induced changes and autophagic flux are accompanied by protein degradation in osteosarcoma cells.

Our data show that the autophagy inhibitors bafilomycin A1 and 3MA, block the 2-ME-mediated conversion of LC3I to LC3II in osteosarcoma cells. While 3MA does not affect osteosarcoma cell growth, bafilomycin had a moderate induction on cell death and enhanced 2-ME-induced cell death (C. Yang, R. Goyal, K.Shogren and A. Maran, unpublished observation). Further work is necessary to evaluate the effect of bafilomycin on osteosarcoma cell death alone and in combination with 2-ME.

The morphological and molecular changes representing autophagosome and autophagic flux are blocked in the presence of PKR inhibition in 2-ME-treated cells. Our studies indicate that RNA-dependent protein kinase (PKR) is essential for the induction of autophagasome formation and the conversion of LC3-I to LC3-II. PKR plays an important role in anti-viral, anti-cancer and anti-proliferative effects. PKR activation in cells leads to phosphorylation of alpha subunit of eukaryotic initiation factor 2 (eIF2α) and subsequent shutdown of viral and cellular protein synthesis [16], [36], [37]. Also, PKR has been shown to induce apoptosis in cancer cells [38], [39]. We have previously demonstrated that PKR plays a role in 2-ME-mediated cell cycle arrest and apoptosis in osteosarcoma cells [24], [25]. Osteosarcoma cells expressing transdominant mutant PKR is resistant to anti-cellular and anti-tumor effects of 2-ME [24]. In this report, we show that 2-ME-mediated autophagosome formation and the conversion of LC3-I to LC3-II are inhibited in cells that express dominant negative mutant PKR protein. PKR has been implicated in the induction of autophagy during virus infection [40]. However, it has not been reported to play a role in drug-induced anti-cancer activities. DRAM (damage-regulated autophagy modulator), is a p53-induced modulator of autophagy and has been implicated in 2-ME-dependent autophagy in Ewing sarcoma cells [41]. 2-ME-mediated induction of autophagy contributes to the cytotoxic cell death in Ewing sarcoma cells [41]. Recently, a few investigations have demonstrated that 2-ME induces both apoptosis and autophagy in different models [41]–[44]. However, the involvement of autophagy in 2-ME-mediated anti-proliferative actions have not been fully understood. The current results demonstrate that both autophagy and apoptosis require PKR protein in 2-ME-treated osteosarcoma cells. Our results show that 2-ME-induces autophagosome and alters LC3-II to LC3-I ratio in wild type PKR expressing cells but not in cells expressing transdominant mutant PKR protein. It is possible that some of the downstream effects contribute to these various actions of PKR. However, further evidence is necessary to determine whether PKR contributes to interplay between autophagy and apoptosis, and that autophagy is required for cell death in 2-ME-treated osteosarcoma cells. Future investigations on molecular candidates associated with autophagy in 2-ME-treated cells could lead to improvement of treatment and development of better anti-cancer strategies in osteosarcoma patients.
